# Integrating assessment of cognitive status in elderly cardiovascular care

**DOI:** 10.1002/clc.23318

**Published:** 2019-12-16

**Authors:** Eiran Z. Gorodeski, Ardeshir Z. Hashmi

**Affiliations:** ^1^ Department of Medicine Case Western Reserve University School of Medicine Cleveland Ohio; ^2^ Harrington Heart and Vascular Institute, University Hospitals Cleveland Medical Center Cleveland Ohio; ^3^ Center for Geriatric Medicine, Medicine Institute, Cleveland Clinic Cleveland Ohio

**Keywords:** cardiovascular disease, cognition, geriatric cardiology, heart failure

## Abstract

Cardiovascular clinicians tend to pay little attention to issues related to cognition, and yet those caring for older adults will encounter a variety of conditions that may lead to cognitive impairment. Most commonly, these include cardiovascular disease‐specific conditions such as cerebrovascular disease or heart failure, but may also include neurodegenerative conditions, mood disorders, medication side effects and polypharmacy, and nutritional deficiencies and metabolic derangements among others. This review presents evidence supporting the importance of assessing cognitive status in older adults with cardiovascular disease, and suggests a practical approach to assessment and management of cognitive impairment in this population when it is found. Special attention is paid to the importance of collaboration between cardiovascular and geriatric specialists, and the value it may bring to patients.

## 1 INTRODUCTION

Cardiovascular clinicians who care for older adults tend to pay little attention to issues related to cognition and cognitive impairment in this patient population. Reasons for this vary and include a lack of awareness about the high prevalence of cognitive impairment in older adults with cardiovascular disease and its association with poor outcomes, a lack of knowledge about how to assess for cognitive impairment, a perception that assessment is lengthy and cumbersome, awkwardness about telling patients they may have problems with cognition, and a lack of understanding of how to manage cognitive impairment when it is identified. This review presents evidence supporting the importance of assessing cognitive status in older adults with cardiovascular disease, and suggests a practical approach to assessment and management of cognitive impairment in this population when it is found.

## 2 COGNITIVE DOMAINS AND DISORDERS

**Figure 1:**
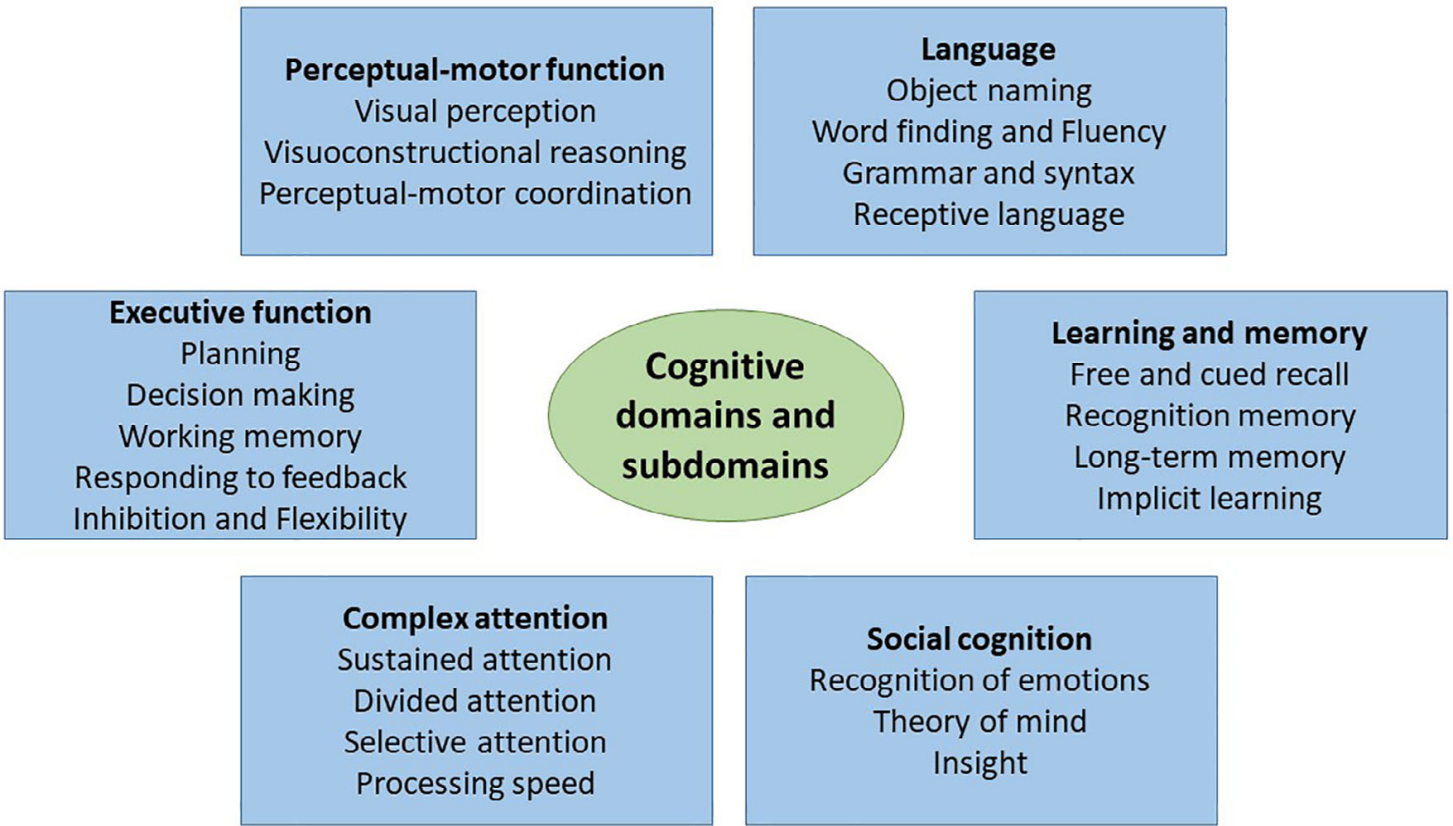
Cognitive domains and subdomains. Adapted from Sachdev et al

In general, a human being's performance is driven by cognitive and noncognitive skills or “domains.” The fifth edition of the Diagnostic and Statistical Manual of Mental Disorders (DSM‐5) defines six key domains of cognitive function—complex attention, executive function, learning and memory, language, perceptual‐motor function, and social cognition—each with its own subdomains (Figure 1).^1^ The DSM‐5 further categorizes cognitive disorders within three separate syndromes, each with a range of possible etiologies, including delirium, mild neurocognitive disorder, and major neurocognitive disorder (or dementia). Noncognitive domains include dexterity, situational awareness, professionalism, compassion, integrity, team work, and resilience—their discussion is beyond the scope of this review.

Mild neurocognitive disorder, a condition commonly found in patients with heart failure (HF), is defined in DSM‐5 as having evidence of modest cognitive decline from a previous level of performance in one or more of the six cognitive domains noted above. To meet criteria for mild neurocognitive disorder, these deficits should not interfere with capacity for independence in everyday activities, and should not be explained by delirium or another mental disorder such as major depression or schizophrenia. Major neurocognitive disorder or dementia, on the other hand, manifests in more severe abnormalities of cognition, whereby the impairment interferes with activities of daily living.

The prevalence of cognitive impairment without dementia has not been clearly defined, though population estimates exist. In the Aging, Demographics, and Memory Study, a longitudinal study of older adults who were representative of the United States population, the derived estimated national prevalence rates of cognitive impairment were approximately 22% in individuals age 71 years or older (16% among ages 71‐79 years, 29% among ages 80‐89, and 39% among ages ≥90).^2^

## 3 COGNITIVE IMPAIRMENT IN CARDIOVASCULAR PRACTICE

Cardiovascular clinicians caring for older adults will encounter a variety of conditions that may lead to cognitive impairment. Most commonly, these include cardiovascular disease‐specific conditions such as cerebrovascular disease or HF, but may also include neurodegenerative conditions (Alzheimer's disease, Lewy‐Body disease, Parkinson's disease, and frontotemporal degeneration), mood disorders (depression and anxiety), medication side effects/polypharmacy, and nutritional deficiencies and metabolic derangements among others.

## 4 VASCULAR COGNITIVE IMPAIRMENT

**Figure 2:**
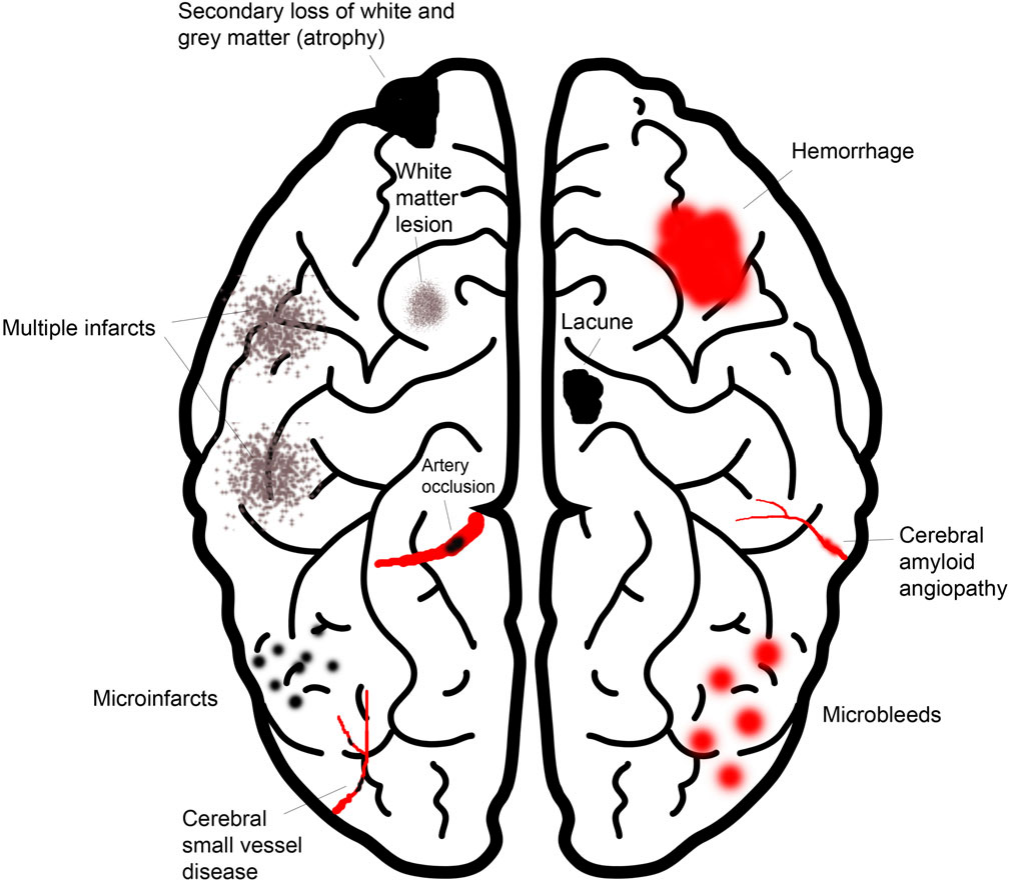
Mechanisms underlying vascular cognitive impairment. Adapted from Reference 4

Vascular dementia is the second most common cause of dementia after Alzheimer's disease, accounting for approximately 20% of cases.^3^ Vascular cognitive impairment is commonly diagnosed by (a) imaging evidence of cerebrovascular disease and a clear temporal relationship between a vascular event (eg, stroke) and onset of cognitive deficits, or a clear relationship between the severity and pattern of cognitive impairment and the presence of diffuse subcortical vascular pathology, and (b) absence of a history of gradually progressive cognitive deficits that may suggest the presence of neurodegenerative disease.^4^ Major mechanisms underlying vascular cognitive impairment (Figure 2) include (a) vascular causes such as large cerebrovascular artery occlusion, cerebral small vessel disease, and cerebral amyloid angiopathy, and (b) brain parenchymal lesions including multiple infarcts of different sizes, white and gray matter loss (atrophy), enlarged perivascular spaces, lobar or deep hemorrhages, and microbleeds.

A tremendous amount of data links vascular cognitive impairment with risk factors familiar to cardiovascular clinicians including increased age, smoking, physical inactivity, obesity, hypertension, chronic hyperglycemia/diabetes mellitus, and dyslipidemia. Additional risk factors include female sex (increased predilection for poststroke dementia), low education level, and possibly some genetic factors.

Vascular cognitive impairment predisposes patients to increased risk of conversion to dementia, institutionalization, and mortality. Occasionally, this population may return to normal cognition particularly when cognitive deficits occurred in context of depression, an acute stroke (approximately ≤20%), HF, or autoimmune disorders.^4^ The mechanism by which the later three medical conditions result in return to normal cognition may be delirium.

## 5 HEART FAILURE

The prevalence of cognitive impairment in HF cohorts is estimated to be 43%, but with a high variability ranging between 30% and 55%.^5^ Reasons for this variability include variation in screening tools used between studies, heterogeneity in HF cause and severity, and differences in inpatient vs outpatient venues of patient care whereby acute illness may cause or exacerbate cognitive problems. Another increasingly recognized reason for variability in estimates of cognitive impairment prevalence is that the burden of cognitive impairment increases cumulatively with HF disease course.^6^, ^7^ In healthy older adults in their 70s, cognitive decline with age is extremely slow, perceptible only on a time scale of decades.^8^ On the other hand, incident HF is associated with a sudden acceleration in prevalence of cognitive impairment. In the Atherosclerosis Risk in Communities Study, cognitive decline over a 15‐year period was greater in participants with HF as compared to those without, even after adjustment for other comorbid conditions.^6^ These observations suggest that pathophysiological mechanisms related to HF drive cognitive impairment over time.

**Figure 3:**
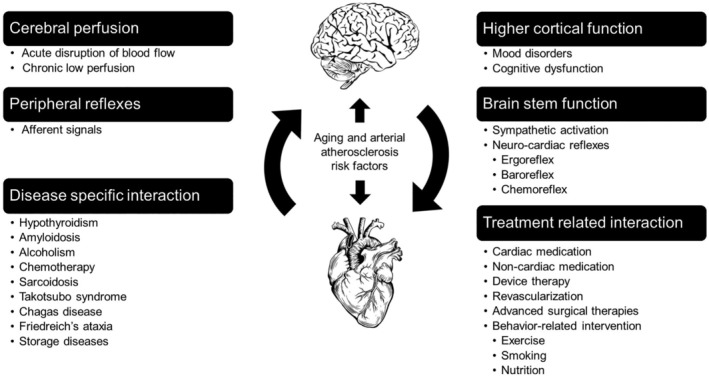
Systematic overview of heart and brain interactions in heart failure (HF). Adapted from Reference 9

The pathophysiology of cognitive impairment in older adults with HF is complex, and often independent of aging. A position paper published by the Heart Failure Association of the European Society of Cardiology in 2018 proposed a novel systematic order of pathophysiological principles, feedback signals, and categories of functional impairment to describe heart and brain interactions in this population.^9^ The underlying principle of this proposed system is the bidirectionality of (a) the failing heart affecting cerebral function and (b) neuronal signals impacting the myocardium.^9^ The five pathophysiological categories proposed (Figure 3) included: (a) impaired cerebral perfusion, (b) impairment of higher cortical function, (c) impairment of brain stem function and peripheral reflexes, (d) treatment‐related interactions, and (e) disease‐specific interactions.

The confluence of factors which leads to cognitive impairment in patients with HF was termed by Havakuk et al,^10^ the “cardiocerebral syndrome,” defined as “a state of cognitive impairment of undefined cause in HF patients, beyond the one anticipated in age‐matched controls, typically accompanied by anatomic brain changes.” Magnetic resonance imaging (MRI) in this population typically shows white matter hyperintensities, particularly periventricular, and gray matter atrophy, particularly involving the hippocampus and frontal cortex. Laboratory studies typically show high levels of neurohormones and inflammatory markers (IL‐6, TNF‐alpha, cortisol, epinephrine). Other medical and neurological conditions that can cause cognitive dysfunction are simultaneously ruled out.^10^

The most common cognitive domain abnormalities among patients with HF are learning and memory, complex attention, and executive function. Examples commonly recognized by HF clinicians include a patient who repeats themselves in conversation often within the same conversation (learning and memory), a patient who cannot keep track of a short list of items (learning and memory), a patient who needs to rely on others to plan activities of daily living or make decisions related to self‐care or medication management (executive function), a patient who has increased difficulty in environments with multiple stimuli including conversation (complex attention), and a patient who has difficulty holding new information in mind such as reporting what was just said (complex attention).

Cognitive impairment in older adults with HF is associated with poor outcomes. Among patients hospitalized for acute decompensated HF, cognitive impairment has been associated with increased readmission risk in multiple studies.^11^ Patel et al^12^ found a nearly 50% 30‐day all‐cause hospital readmission risk in those older adults hospitalized for HF who had cognitive impairment vs approximately 25% risk in those who did not have cognitive impairment. An important observation by Agarwal et al^13^ in another of these studies was that fewer than 9% of patients with cognitive impairment had it documented in their medical record, highlighting the need to change practice. Cognitive impairment is now recognized as an important risk‐adjust marker of hospital readmission risk in older adults hospitalized for HF.

Among community‐based older adults with HF, cognitive impairment has complex cross‐sectional associations with physical frailty, sleep disorders, and mood disorders including depression, anxiety, and hopelessness.^14^, ^15^, ^16^, ^17^ Cognitive impairment has been linked to a variety of poor outcomes including worse health‐related quality of life, increased spousal/caregiver distress, increased disability, worse cardiovascular outcomes at 180 days, and increased mortality risk.^18^, ^19^ A common link, which has immense practical implications for clinicians caring for older adults with HF, is that cognitive impairment in this population is associated with worse self‐management, self‐care, and self‐confidence.^20^ A high index of suspicion is warranted as patients with cognitive impairment are less likely to seek assistance.^20^

### 5.1 When should clinicians screen for cognitive impairment?

Little is known about the ideal settings and time points during which screening for cognitive impairment in older adults with cardiovascular disease is indicated. Based on our observations and practice, we believe that such screening may be valuable (a) the first time a cardiovascular clinician meets a new patient in the outpatient setting, (b) longitudinally across time in the outpatient setting, and (c) during a hospitalization and prior to discharge back to the community.

First time appointments in the outpatient setting tend to be longer than established visits. The extra time is important to allow the clinician to get a more comprehensive understanding of their patient. Gorodeski et al described a “domain management approach” to older adults with HF that calls for evaluating patients in four domains: medical, physical function, mind and emotion, and social environment.^18^ A key component of the mind and emotion domain is evaluation of cognition, which we believe should be done in various ways during the history gathering and as part of the physical exam (further discussion below). Establishing a baseline understanding of patients' cognition is important for several reasons. First, it may give clinicians a sense of whether the patient has the ability to understand complex explanations. Second, it establishes a baseline as disease course and treatments can impact cognition over time. Third, it will alert clinicians about risk of poor self‐management skills, and will therefore allow clinicians to leverage resources such as the patient's social circle or other community‐based services. Finally, the presence of cognitive impairment on a screening test may suggest the presence of coexisting neurocognitive conditions that may warrant referral to appropriately chosen consultants.

Intermittent brief screens of cognition may bring utility in longitudinal outpatient care as well. This may be especially relevant in patients who are seemingly “noncompliant” with following medical directions, demonstrate poor self‐care, or are recurrently admitted to the hospital for decompensation of disease. Not infrequently issues related to impaired executive function, problem solving, and memory may explain these poor outcomes. Additional consideration should be given to screening these patients for mood disorders including depression, and sleep disorders including obstructive and central sleep apnea. More detailed discussion of these issues is beyond the scope of this review.

Hospitalization for acute HF exacerbation is a sentinel event for patients with chronic HF, and carries poor prognostic implications. While the pathophysiology of HF exacerbation (congestion, poor forward flow, arrhythmias, etc.) can clearly contribute to cognitive impairment, stressors related to the hospitalization itself may contribute as well. In his landmark perspective titled “Post‐Hospital Syndrome—An Acquired, Transient Condition of Generalized Risk,” Dr Harlan Krumholz noted: “During hospitalization, patients are commonly deprived of sleep, experience disruption of normal circadian rhythms, are nourished poorly, have pain and discomfort, confront a baffling array of mentally challenging situations, receive medications that can alter cognition and physical function, and become deconditioned by bed rest or inactivity. Each of these perturbations can adversely affect health and contribute to substantial impairments during the early recovery period, an inability to fend off disease, and susceptibility to mental error.”^21^ Among older adults hospitalized for HF cognitive impairment is associated with post‐discharge readmission and mortality risk.^12^, ^13^ For these reasons, screening for cognitive impairment in the inpatient setting is important. The ideal timing to conduct this screening is unknown, but is likely most valuable when conducted as part of discharge planning, approximately 2 to 3 days prior to the patient's transition from hospital to community.^12^

**Figure 4:**
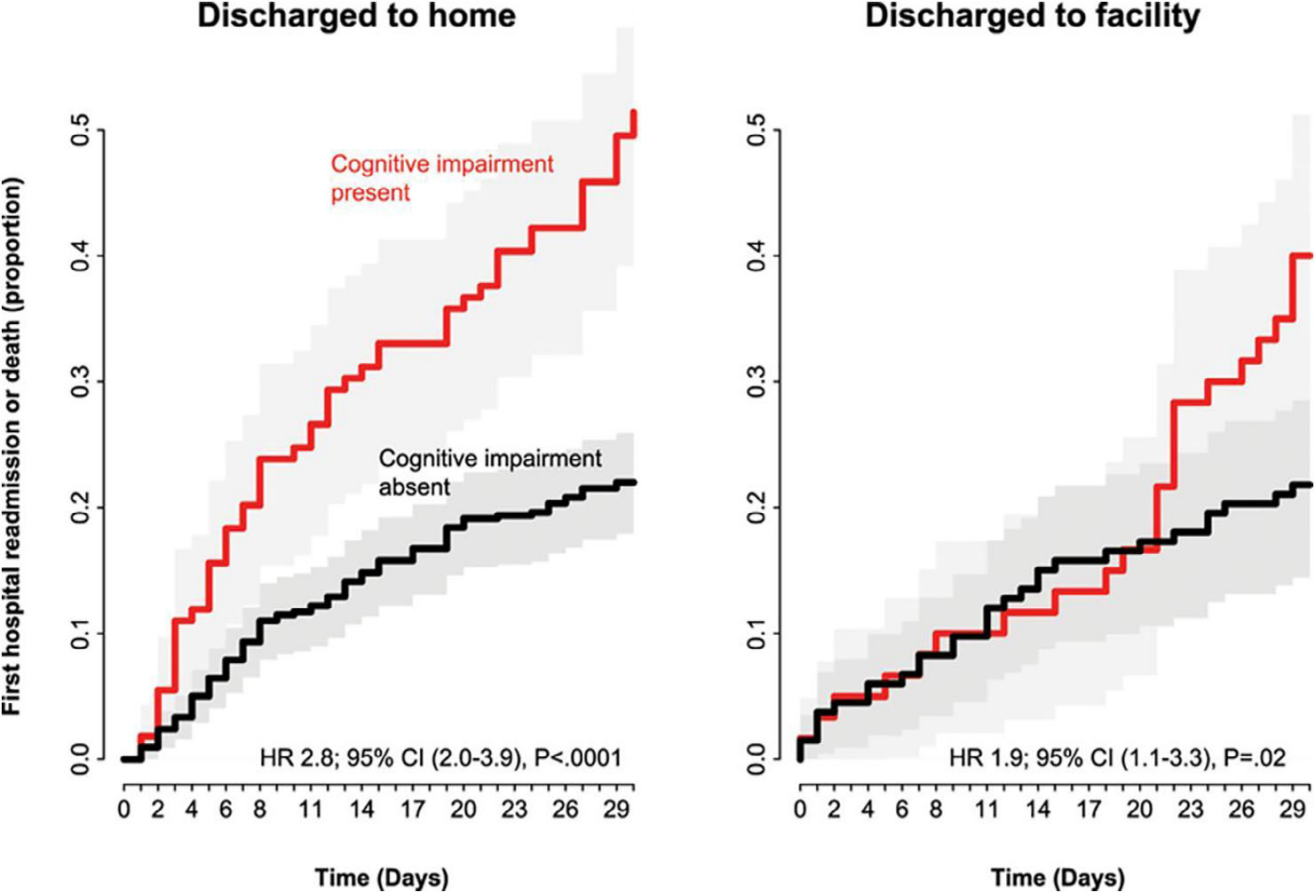
Cognitive impairment and postdischarge outcomes among older adults hospitalized for heart failure, stratified by venue of discharge. Published with permission from Reference 12

In one study of cognitive impairment and postdischarge outcome in older adults with HF, there was effect modification by venue of discharge, whereby patients with cognitive impairment discharged to a nursing facility had longer time to hospital readmission or mortality as compared with those discharged home (Figure 4).^12^ This suggests that for patients with cognitive impairment structured postdischarge programming offered at nursing facilities may protect from worse outcomes. Randomized clinical trials are needed to assess whether this observation is valid. Further, it is unknown whether structured home care programs specifically designed for older adults with HF and cognitive impairment can improve outcomes.

### 5.2 Screening for cognitive impairment: Tools and approaches

**Figure 5:**
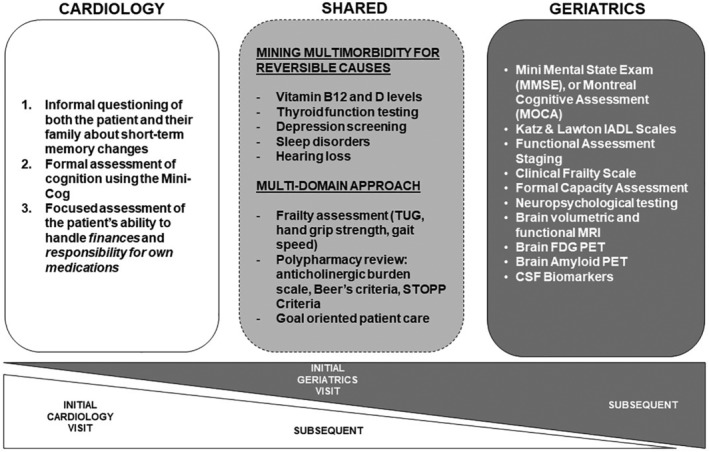
Screening for cognitive impairment: tools and approaches

There is no formal consensus about which tools should be used to assess cognition in older adults who have cardiovascular disease. We believe that the following three tools/approaches are of value (Figure 5): (a) informal questioning of both the patient and their family about short‐term memory changes, (b) formal assessment of cognition using the Mini‐Cog, and (c) focused assessment of the patient's ability to handle *finances* and *responsibility for own medications*, preferably using the finances and medications domains of the Lawton instrumental activities of daily living (IADL) scale.^22^

Subjective memory complaints in older adults are important but are often missed or overlooked in most clinical encounters, both in specialist and primary care settings.^23^, ^24^, ^25^, ^26^ Clinicians should take note of subjective short‐term memory changes—especially those reported by family members—as it has long been recognized that these observations may have a high level of specificity for pathological cognitive changes including dementia. Patients themselves may not be able to fully recollect the true extent of their short‐term memory changes, or may withhold this information from their clinicians fearing loss of independence, self‐esteem, or the possibility of more testing. Focusing on short‐term memory changes is important as long‐term memory is preserved in most dementias.

The Mini‐Cog, a three‐word recall and clock‐drawing test, is a validated screening tool for cognitive impairment.^27^ It is ultrashort and takes less than 3 minutes to complete, making it is ideal for use in busy clinical settings.^28^ The Mini‐Cog, and especially abnormal clock drawing, make cognitive impairment visible even if it has not yet become apparent to patients and families. While a positive Mini‐Cog finding does not “rule in” dementia, it enables appropriate navigation toward referral to a specialist such as geriatricians for further cognitive evaluation. The Mini‐Cog can also be easily integrated into the clinical workflow of medical assistants' vital screening and triage process, ahead of the cardiovascular clinician's arrival into the exam room.

IADLs include using the telephone, grocery shopping, cooking, housekeeping, laundry, managing medications and finances, and driving. Progressive compromise of these tasks may be related to underlying cognitive impairment, and is associated with loss of functional independence.^29^ Arguably, the IADL components that are most dependent on cognitive skills are medication self‐management and ability to handle finances.^30^ The Lawton IADL scale^22^ details an approach to assessment of these two domains as follows. Failures of the medication self‐management domain are defined as a patient who depends on others to prepare medication dosages in advance and/or is not capable of dispensing own medication. Failure of the finances domain is defined is a patient who is incapable of handling money. Cardiovascular clinicians should pay close attention to these failures if present, as they may suggest presence of cognitive impairment.

## 6 SCREENING FOR POTENTIALLY REVERSIBLE CAUSES OF COGNITIVE IMPAIRMENT

Because cognitive impairment in older adults with cardiovascular disease may be multifactorial, it is important for cardiovascular clinicians to be aware of nonneurological/noncardiac medical causes and how to screen for them. These causes can help inform immediate and time efficient next steps in cognitive diagnostic evaluation. Furthermore, these factors are not only easily identifiable but also readily reversible. These include thyroid dysfunction, vitamin B12 deficiency, and depression. More recently, there is evidence for underlying sleep disorders,^31^ sensorineural hearing loss,^32^ and vitamin D deficiency^33^ as potential treatable contributors to cognitive impairment. Harnessing brief screens such as the STOP‐BANG Sleep Apnea questionnaire,^34^ Hearing Handicap Inventory for the Elderly Screening Version,^35^ and the brief Geriatric Depression Scale^36^ may help in screening for these causes. Blood testing for thyroid function, vitamin B12, and vitamin D can be ordered even prior to specialist referral to facilitate care.

### 6.1 Barriers to cognitive status assessment

Despite the fact that screening for cognitive impairment among older adults with cardiovascular disease is intuitive, valuable, and easily accomplished, little research exists about barriers to implementation. In one survey of family physicians and general practitioners from Canada, only 24% of practitioners routinely screened patients for cognitive impairment, although 82% believed screening was needed.^37^ The most common perceived barriers to screening were lack of time (82% of respondents; each respondent could list more than one barrier), patients offended or resistant about being screened (58%), negative consequences of follow up (24%), lack of proven benefit of treatment for cognitive impairment/dementia (22%), and available screening tests inadequate (22%).

Tools exist to facilitate screening of cognition, even among clinicians who have little to no formal training in this area. As noted above the Mini‐Cog is an ideal screening tool for busy clinical practices, as it takes less than 3 minutes to administer. Learning how to deploy the Mini‐Cog correctly is essential, and some have noted that the standardized instructions for clinicians may be confusing and lead to errors in administration and scoring. To address this, Tam et al previously developed graphical instructions for administration and scoring the Mini‐Cog (https://mini-cog.com/graphical-mini-cog/), essentially a double‐sided pocket card with cartoon instructions and a simple scoring algorithm. Use of these graphical instructions vs standardized instructions was tested in a randomized clinical trial which found that among registered nurses without prior training in Mini‐Cog, use of the card increased the accuracy and speed of test administration.^38^

A potentially promising future direction to address barriers to cognitive impairment screening is the use of technology, and specifically a tablet device that can be portable, eliminates the need for a trained administrator, facilitates standardization of assessment, allows automated scoring, and provides integration of results directly into the electronic medical record. Gorodeski et al^39^ studied the use of the Processing Speed Test, a test of information processing, attention, and working memory, administered on an Apple iPad in older adults hospitalized for HF. In this proof‐of‐concept work, the investigators found that tablet‐based deployment of a cognition test was feasible, reliable, and valid, suggesting a future state where cognitive impairment screening can occur entirely independent of any clinician.

### 6.2 Responding to cognitive impairment: Practical considerations

Cardiovascular clinicians should adopt a common approach to management of geriatric syndromes in older adults with cardiovascular disease that includes: (a) awareness, (b) screening, (c) incorporation into decision‐making, and (d) leveraging team‐based care by referring to and collaborating with appropriate colleagues. These approaches have been discussed in detail elsewhere as they relate to older adults with cardiovascular disease and HF.^18^

A nuanced issue related to cognitive impairment and cardiovascular care that clinicians will encounter are the concepts of informed consent and capacity. The presence of cognitive impairment on screening tests does not automatically imply that a patient is unable to make their own decisions regarding their care. Decision‐making capacity is contingent upon a patient (a) understanding the risks vs the benefits of a proposed treatment, (b) being able to relate back the ramifications of scenarios that may carry the risk of harm, and (c) exhibiting logic and coherence in relating back what would be the steps needed to counteract such an unsafe situation.^40^ Depending on the situation encountered cardiovascular clinicians may or may not need to collaborate with specialists such as geriatricians.

## 7 WHAT TO EXPECT FROM A GERIATRICS CONSULTATION

While robust cognitive evaluations are offered by a variety of specialists including neurologists and psychiatrists, geriatricians add unique value via their holistic view of the older adult. A geriatrics consultation allows an in depth multimodality cognitive evaluation, usually including more extensive tests of cognition (eg, Montreal Cognitive Assessment or the Mini Mental Status Examination), evaluation for potentially reversible medical causes of memory impairment including polypharmacy, assessment of behavioral manifestations of cognitive impairment and dementia, and evaluation of function and delineation of frailty. Complex neuroimaging including brain MRI and PET scan may be considered. For scenarios in which diagnostic uncertainty remains, neuropsychological testing with several hour‐long composite battery of cognitive tests may be completed. Geriatricians extensively focus on patient values (“what matters most”), caregiver stress, and potential transitions to higher levels of community‐based care such as continuing care retirement communities (including Independent and Assisted Living) and nursing facilities.
